# Opening of the Colleges

**Published:** 1896-09

**Authors:** 


### BUFFALO MEDICAL JOURNAL.

A Monthly Review of Medicine and Surgery.

*EDITORS:*

THOMAS LOTHROP, M. D. - - WM. WARREN POTTER, M. D.

All communications, whether of a literary or business nature, should be addressed to the managing editor: 284 FRANKLIN STREET, BUFFALO, N. Y.

VOL. XXXVI. SEPTEMBER, 1886. No. 2.

### OPENING OF THE COLLEGES.

THE month of September generally marks the beginning of the medical academic year; at least we believe the majority of medical colleges are thrown open for work some time during this month, though they for the most part select the latter part thereof in which to begin the year's curriculum. At all events, before the next issue of the JOURNAL the majority of the medical colleges east of the Alleghany mountains will be under full headway.

It is a matter of extreme congratulation on the part of the friends of improvement in medical education that most of the colleges within the territory named have established a four years' course of medical study, and this, too, in most instances, in advance of any statutory requirements making this standard authoritative. This is in striking contrast to the condition that existed ten years ago, or even less, when these same schools, for the most part, demanded but two years' collegiate instruction upon which to issue a diploma.

The extension of time by no means represents the only advance that has been made in medical college training. Clinical instruction has been pushed forward to the first place in the curriculum of medical teaching, laboratories have been established, in which chemistry, physiology, biology, bacteriology and other branches of the abstract sciences are taught; recitation courses have been instituted; demonstrations by manikins, drawings, plates and casts and in the anatomical room have been further perfected; clinical teaching of midwifery has been insisted upon, and didactic teaching has been relegated from the place of first importance that it formerly held to a position subordinate and quite secondary to the foregoing category. These and other improvements in medical teaching, not necessary to mention here in detail, are such as to accentuate the last decade of the nineteenth century as the most progressive period in the history of medicine, and to lay the foundation for an auspicious beginning of the next century of medical history.

No careful observer will attempt to deny that the medical examining and licensing boards in the several states have played an important part in bringing about many of these reforms ; nor can it be disputed that they will continue to exercise a constantly increasing influence in advancing the standards of medical teaching as time moves forward. This, too, notwithstanding the fact that the finger of derision is pointed at them, or the stings of criticism are leveled at their work, even from sources that could not have been anticipated, and whose attempts at weakening their influence might better be directed toward strengthening their efforts to elevate the standard of American medical teaching and to make the American diploma respected at home and abroad.

It is interesting, however, to note that these criticisms for the most part come from a few exceptional men who have a gift for loud speech, and fancy themselves aggrieved because they cannot overstep the statutory provisions with a seven league boot stride, defy all attempts at restraint and freely roam about the country plying the practice of their specialties at watering places or summer homes, regardless of state boundary lines or other geographical limits, to their towering hopes or soaring ambitions.

It is our special purpose, however, at this time to point to another side of this question and to which we will now address ourselves. In order that students may have a full understanding of the state control of medical practice and gain a proper respect for the salutary provisions of medical police laws, we think it would be wise for medical faculties to designate at least one of their number to address their students, either at the opening of the college course or at an appropriate time during its progress, on this subject. Whenever there is a radical change in a system that affects a considerable number of intelligent persons, as is the case with the introduction of state medical examinations for license to practise medicine, it is important that those most interested by such change shall be made acquainted with all the facts connected with it. Whenever this has been carefully done the result has been beneficial.

Two years ago Prof. F. Whitehill Hinkel, M. D., of Buffalo, chose as the title of an introductory address to the forty-ninth course of lectures at the medical department of the University of Buffalo, "A review of the relations between the state and the medical profession in New York." This address was a careful analysis of the present status of these relations, and surely did not fail to exercise a useful influence over the classes in that college during the academic year in which it was delivered. Again, Prof. Willis G. Tucker, M. D., of Albany, chose as the subject of his opening address to the Albany Medical College course last year, "State control of medicine." He, too, considered this subject in great detail and explained it fully to his hearers. The effect of such instruction cannot be otherwise than good. It tends to increase the respect of the student for the laws pertaining to education and contributes to the efficiency of the school in which he is receiving instruction. Moreover, it also serves to create that discipline which should be as much a part of medical teaching as of other collegiate instruction.

The two medical colleges in Buffalo each has wisely entered upon a four years' course, which has been heretofore announced in these columns and which, too, has been distinctly set forth in their annual announcements lately published. It is, however, a matter of comment that but one medical college in the state of New York —Syracuse University—is a member of the Association of American Medical Colleges. Now that most of them have adopted four years' courses it is to be hoped that they will unite with this association.

Finally, it may be remarked that the outlook to the friends of improvement of medical education in this country is most encouraging. The steadfast support given to state control in medicine by every reputable magazine in the country, the unity of feeling and action between medical colleges and state medical examining boards, and the healthy atmosphere surrounding the field and everywhere permeating its furrows and corners, bespeaks an auspicious beginning for the history of American medicine that will be written in the twentieth century.

